# A Rare Case of Scrotal Swelling: Prostate Cancer Metastasis Masquerading as a Complicated Hydrocele

**DOI:** 10.7759/cureus.74773

**Published:** 2024-11-29

**Authors:** Alexandra S Robinson, Zakaria W Shkoukani, Rauf Khadr, James Stevenson, Mohamed I Abdulmajed

**Affiliations:** 1 Department of Urology, Mersey and West Lancashire Teaching Hospitals NHS Trust, Liverpool, GBR

**Keywords:** hydrocele, lymphovascular invasion, perineural invasion, prostatic adenocarcinoma, testicular metastasis

## Abstract

Metastasis of prostate cancer to the testes is exceptionally rare. We report the case of a 67-year-old male with a 10-year history of high-risk prostate cancer, previously treated and currently in remission, who presented with left scrotal swelling. The swelling was clinically and radiologically diagnosed as a hydrocele and treated surgically. A postoperative localized infection complicated the hydrocele repair. Two years after the surgery, the patient presented with a reoccurrence of scrotal swelling, coinciding with an insignificant increase in serum prostate-specific antigen (PSA) levels from 0.4 ng/mL to 2.0 ng/mL. Furthermore, computed tomography (CT) imaging of the abdomen and pelvis demonstrated no suspicious masses and normal appearance of the underlying testes. However, repeat ultrasonography of the left testis revealed an irregular and diffusely heterogeneous testis with increased vascularity. Presuming these findings to be fibrotic scrotum following a hydrocele repair complicated with postoperative infection, a left inguinal orchidectomy was performed. Histopathological analysis revealed extensive infiltration of the testicular parenchyma by adenocarcinoma, characterized by cribriform glands, round nuclei, and prominent nucleoli. Immunohistochemical analysis revealed widespread positivity for PSA and moderate, patchy expression of NKX3.1. Additionally, there was focal, strong staining for chromogranin and synaptophysin. A collaborative evaluation by the multidisciplinary team involving urological surgeons, pathologists, and radiologists was crucial in reaching the final diagnosis of metastatic prostate adenocarcinoma to the testis. This case emphasizes the importance of maintaining a high suspicion for metastasis in prostate cancer patients, even when clinical or radiological findings are not prominent, as the diagnostic approach may not always follow a predictable course.

## Introduction

Prostate malignancy remains a significant global health issue, representing the most commonly diagnosed malignancy in men and accounting for approximately 15% of all cancer cases worldwide [1,2]. Due to shifting demographics and increasing life expectancy, the global incidence of prostate cancer is projected to rise from 1.47 million cases in 2020 to approximately 2.9 million by 2040. It is one of the leading causes of cancer-related mortality among men, second only to lung cancer [3]. Metastatic prostate adenocarcinoma is associated with a poor prognosis, with a five-year survival rate of 30.5% [4]. Common metastatic sites include local and distant lymph nodes, the axial skeleton, lungs, liver, and rarely the brain. Local node metastasis typically involves pelvic nodes (obturator, iliac, presacral), while distant nodes include para-aortic and caval lymph nodes [1,5]. Metastasis to the testes occurs in approximately 0.3-0.5% of cases [6]. Such metastases are often discovered incidentally during procedures like orchidectomy or autopsy, contributing to delayed diagnosis and limited opportunities for early intervention [5]. Herein, we report a rare case of metastatic prostate cancer, unexpectedly identified during testicular ultrasonography of an initially presumed fibrotic scrotum following a hydrocele repair complicated with postoperative infection.

## Case presentation

A 67-year-old male initially presented to the urology clinic with lower urinary tract symptoms (LUTS), including poor flow, hesitancy, incomplete bladder emptying, and nocturia. Their past medical history included atrial fibrillation, and he was taking apixaban. His symptom assessment and routine blood tests, including full blood count and kidney and liver function tests, revealed no significant abnormalities. The prostate-specific antigen (PSA) level was 3.6 ng/ml. Digital rectal examination (DRE) identified an irregular nodularity of the prostate. He, therefore, underwent prostatic biopsies, a multiparametric MRI of the prostate, and a bone scan. Investigations concluded a high-risk prostatic adenocarcinoma (radiological staging of T3bN0M0; histological Gleason score of 4+5). He underwent successful radical treatment, including three years of androgen deprivation therapy with six-monthly Decapeptyl injections. Additionally, he participated in the STAMPEDE trial, receiving abiraterone, enzalutamide, prednisolone, and external beam radiotherapy (72 Gy in 32 sessions) to the prostate and pelvis. Abiraterone and enzalutamide were shortly stopped after three cycles due to liver toxicity. He was thereafter followed up with PSA surveillance whilst in remission (PSA nadir of 0.1 ng/mL).

**Figure 1:**
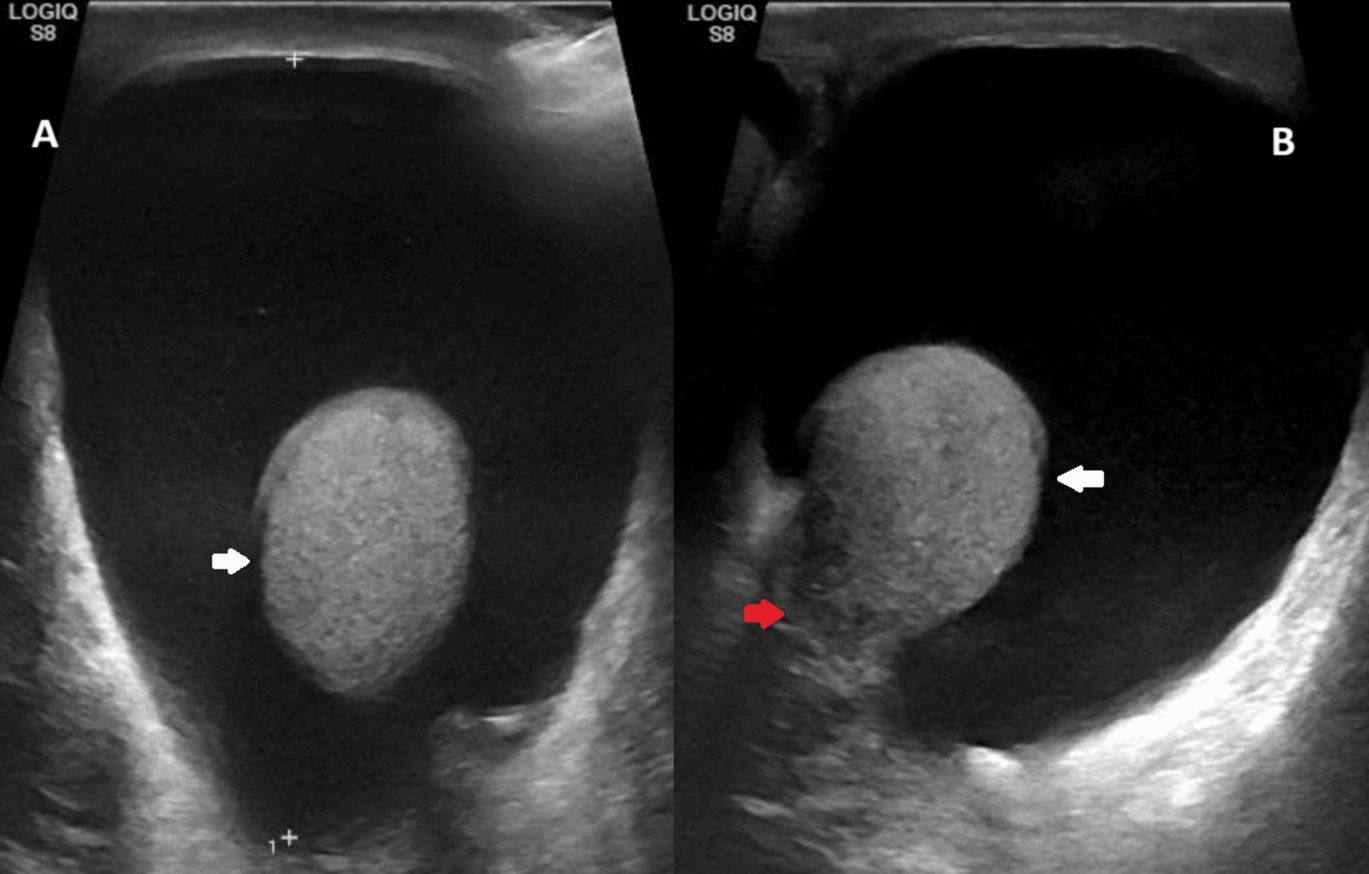
Ultrasonographic imaging of the left testis with hydrocele (A): Ultrasonography of the left testis demonstrates a large anechoic (dark or black) fluid collection displacing the testis (white arrow) inferiorly, with the testis appearing normal and homogeneous in echotexture. This confirmed the most likely differential of a hydrocele. (B): Ultrasonography of the left testis shows the same hydrocele with normal, homogeneous echotexture of the testis (white arrow) and a normal-appearing epididymis (red arrow).

The patient was re-presented to the urology clinic seven years later with new left-sided scrotal swelling. Clinically, the swelling was consistent with a hydrocele, confirmed on ultrasound as an anechoic fluid collection surrounding a normal, homogeneous testis within the tunica vaginalis (Figures 1A, 1B). As he was otherwise asymptomatic, he opted for conservative (non-surgical) management.

One month later, the patient returned to the clinic with further scrotal enlargement and associated discomfort, leading to the decision to proceed with surgical intervention. He underwent hydrocele repair under a general anesthetic. Surgical notes from the procedure documented that the tunica vaginalis was incised, with clear fluid evacuated, exposing a macroscopically normal testis. The procedure involved the excision of a thickened tunica vaginalis followed by plication using 2/0 Vicryl sutures, confirming a plication of the hydrocele. Postoperatively, the patient developed a localized infection, presenting with fever, scrotal swelling, and purulent discharge from the surgical wound. Blood cultures yielded no growth. The infection was confirmed via ultrasound, demonstrating scrotal wall edema and inflammation but no collections. The infection was managed conservatively with an initial course of intravenous antibiotics and transitioned to oral antibiotics. The patient achieved full recovery with satisfactory wound healing. Three months later, a further review in the clinic confirmed the resolution of the infection and swelling, leaving a firm left hemi-scrotum on physical examination, consistent with postoperative and post-infection changes. PSA surveillance continued, measuring 0.4 ng/mL at the time of hydrocele repair.

**Figure 2:**
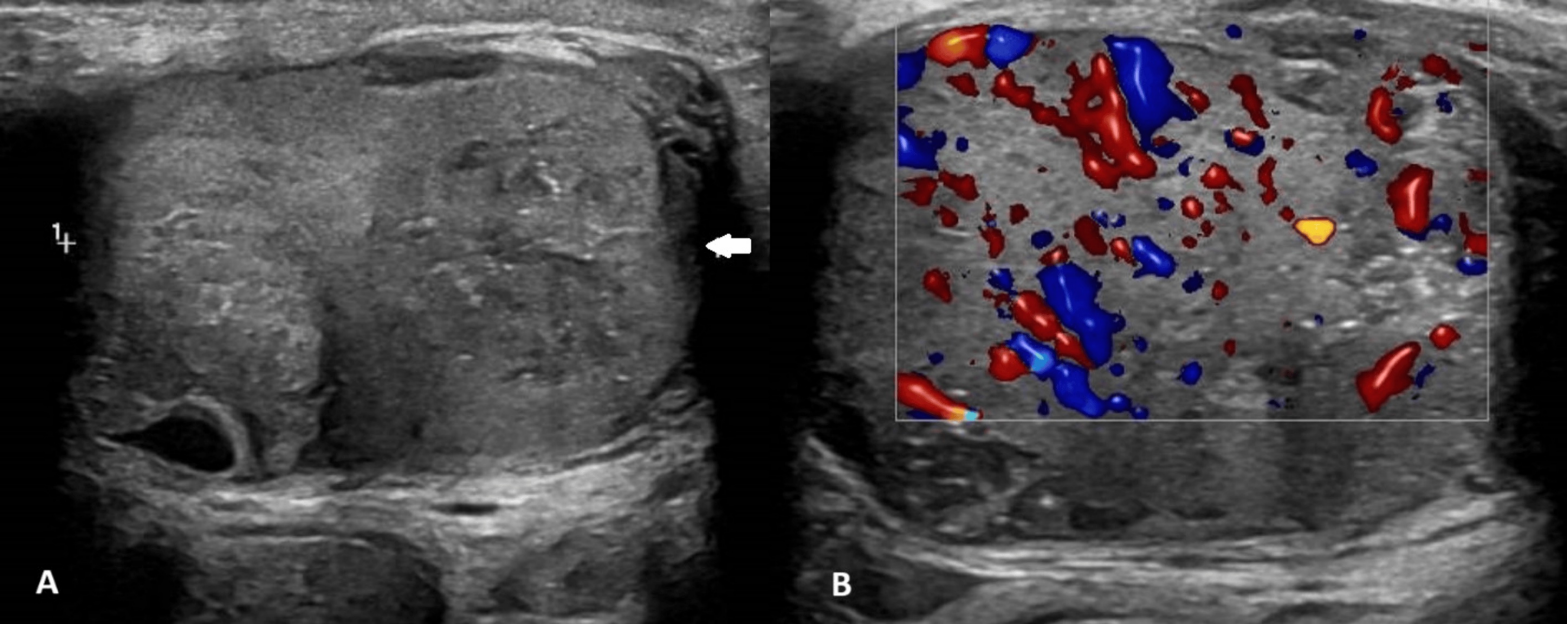
Repeat ultrasonography of the left testis (A): Grayscale ultrasonography of the left testis shows an irregular contour with diffuse heterogeneous echotexture (white arrow), indicating possible parenchymal changes. Multiple small calcifications are visible in the lower pole region. The left epididymis is not adequately visualized in this view, and no hydrocele is present. (B): Color Doppler ultrasonography of the left testis reveals increased vascularity throughout the testicular parenchyma, suggesting hyperemia associated with inflammatory or neoplastic processes.

Two years later, the patient presented as an emergency with suspected left ureteric colic, and CT imaging of the abdomen and pelvis suggested the recent passage of a small ureteric stone, with no other significant findings. Concurrently, his PSA levels exhibited a gradual rise to 2.0 ng/mL, and he incidentally reported a recurrence of the left testicular swelling. Repeat testicular ultrasonography was therefore performed, demonstrating significant changes in the left testis, including an irregular contour, diffuse heterogeneity of echotexture, increased vascularity, and the presence of calcific foci in the lower pole (Figures 2A, 2B). These findings were reviewed at the multidisciplinary team (MDT) meeting. Tumor markers did not suggest primary testicular cancer, with an alpha-fetoprotein (AFP) level of 6, a human chorionic gonadotrophin (β-HCG) level of <3, and a lactate dehydrogenase (LDH) level of 192. Due to diagnostic uncertainty and significant scrotal swelling and pain affecting patients' quality of life, a left inguinal radical orchidectomy under general anesthesia was performed. The preoperative examination revealed an abnormal testicular consistency, described as firm to hard, despite a normal size. Notable findings included significant scarring and adhesion to the overlying dartos muscle layers, indicative of prior infection.

**Figure 3:**
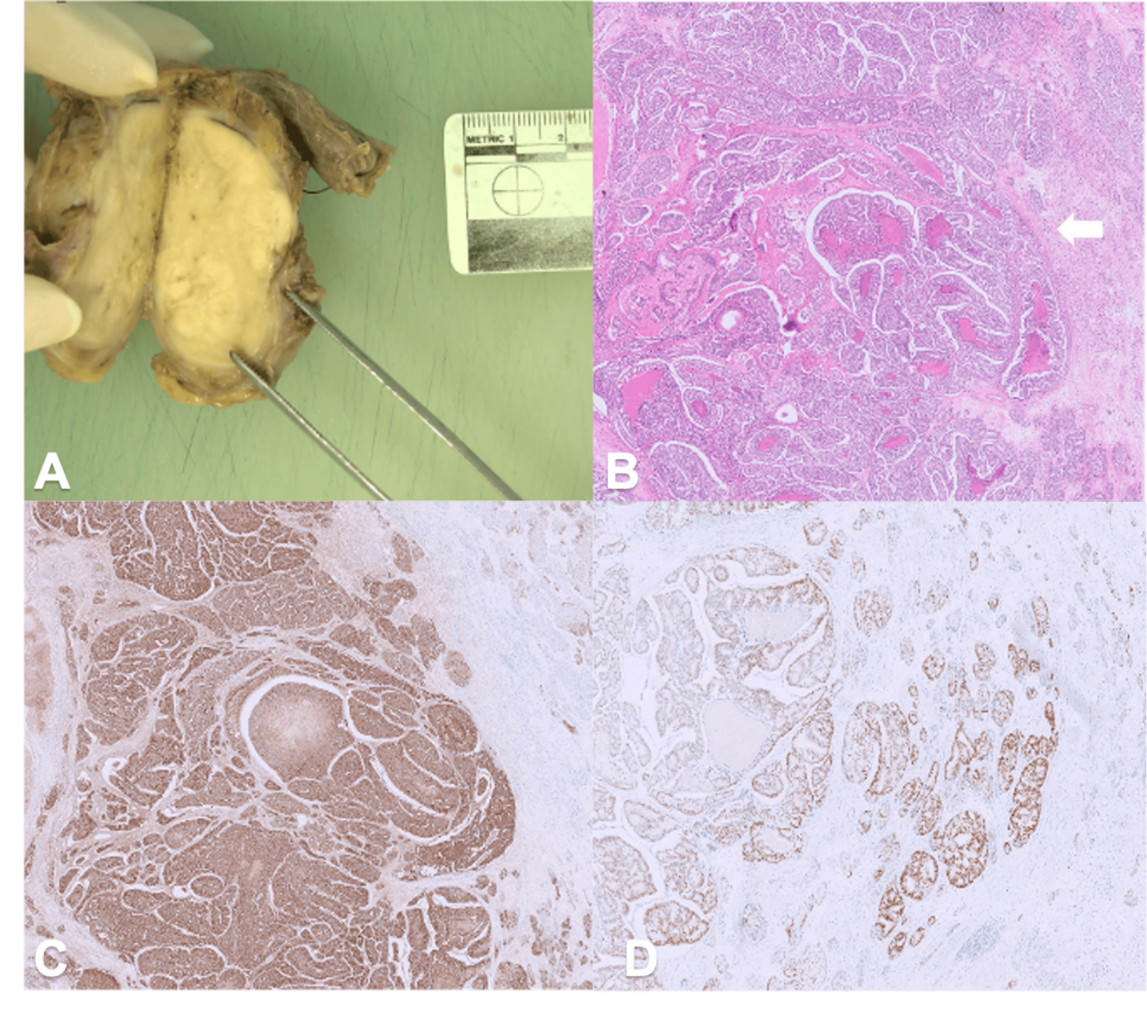
Macroscopic, histopathological, and immunohistochemical findings of the orchidectomy specimen (A): Macroscopic appearance of the bisected post-fixation orchidectomy specimen, revealing near-total replacement of the testicular parenchyma by a tan-white, solid tumor mass. (B): Hematoxylin and eosin-stained section showing the microscopic architecture of the adenocarcinoma (white arrow), with features consistent with Gleason score 4+4 prostatic adenocarcinoma. The tumor exhibits cribriform and fused glandular patterns indicative of high-grade malignancy (10x magnification). (C): Immunohistochemistry for prostate-specific antigen (PSA) in a section from the same paraffin block, demonstrating positive cytoplasmic staining, confirming the tumor’s origin as prostatic adenocarcinoma (10x magnification). (D): Immunohistochemistry for NKX3.1, a prostate-specific marker, also shows positive nuclear staining, further supporting the diagnosis of metastatic prostatic adenocarcinoma in the testis (10x magnification).

Macroscopic examination of the specimen revealed that the testis was largely replaced by a cream-white solid mass with areas of necrosis, abutting but not extending beyond the tunica albuginea (Figure 3A). Histologically, the tumor exhibited cribriform glandular structures with round nuclei and occasional prominent nucleoli, confirming extensive infiltration of the testicular parenchyma by adenocarcinoma (Figure 3B). Evidence of lymphovascular and perineural invasion was identified, although the spermatic cord margin remained clear. Immunohistochemical analysis demonstrated diffuse positivity for PSA (Figure 3C), moderate patchy expression of NKX3.1 (Figure 3D), and focal strong positivity for chromogranin and synaptophysin, consistent with metastatic prostate adenocarcinoma. The neoplastic cells were negative for OCT3/4, CD117, CD30, AFP, inhibin, calretinin, GATA-3, and CDX2. The final diagnosis was, therefore, metastatic adenocarcinoma of the prostate involving the left testis.

Postoperatively, the patient’s PSA levels decreased significantly, from 2.0 ng/mL to <0.2 ng/mL, indicating a favorable response to surgical intervention. Further contrast-enhanced staging CT scans of the chest, abdomen, and pelvis, as well as bone scintigraphy, showed no evidence of further metastatic disease. Following further MDT discussions, he was referred back to the oncology team and has since been commenced on androgen deprivation therapy (six-monthly Decapeptyl injections), and his PSA remains undetectable (<0.2 ng/mL) for the last four months whilst on treatment.

## Discussion

Metastasis of prostate adenocarcinoma to the testis is an uncommon pattern of disease dissemination and is typically identified incidentally through imaging [7-12]. Isolated testicular involvement occurs in only 15-20% of cases, while the majority (80-85%) involve metastases to the testis alongside other organs, such as bones, lymph nodes, lungs, or liver. Notably, prostate cancer accounts for approximately 15% of secondary testicular neoplasms, making it the most common primary source [1]. Testicular metastasis is typically identified 5-10 years after the initial diagnosis of prostate cancer, with a reported range of 1 to 18 years [6]. Approximately 150 cases of testicular metastasis from prostate cancer have been reported in the literature according to existing studies [1,4,7-15]. Notably, only a few instances have described metastatic prostate cancer masquerading as a unilateral hydrocele, highlighting the unique nature of this presentation [13-15]. This case emphasizes the importance of maintaining a high index of suspicion for malignancy in patients with a history of prostate cancer who develop new scrotal abnormalities.

Several mechanisms for testicular invasion by prostate cancer have been proposed in the literature. One possibility is the direct extension of the tumor into adjacent structures, such as the testes, mainly when the tumor arises in prostate regions near the seminal vesicles or pelvic wall. Alternatively, prostate cancer frequently metastasizes through lymphatic channels, and invasion of the lymphatic system may facilitate malignant spread to the testes [16,17]. Other suggested routes include hematogenous dissemination, peritoneal spread, or direct contamination during surgery [16], though these mechanisms are less supported. Direct extension and lymphatic spread remain the most widely accepted pathways of metastasis [1,4]. However, in our case, testicular metastasis likely occurred via the hematogenous spread, as the tumor did not involve the tunica albuginea, excluding a direct extension.

The management of primary testicular tumors differs markedly from testicular metastases due to their distinct etiologies and disease patterns. Primary testicular tumors, such as germ cell tumors, are typically treated by surgical excision, followed by either chemotherapy or occasionally external beam radiotherapy, depending on the tumor type and stage [1,4,14]. In contrast, testicular metastases, which originate from malignancies elsewhere (including prostate cancer), are primarily managed through systemic therapies directed at the primary pathology. This approach includes hormonal manipulation therapy, chemotherapy, or targeted radiotherapy rather than direct surgical intervention on the testis [11]. In cases where testicular metastases cause significant discomfort, pain or swelling, palliative surgery or radiotherapy could be considered. However, these symptoms are typically secondary to the systemic treatment aimed at controlling the primary malignancy [4,13]. In this particular case, postoperative staging CT and bone scintigraphy showed no evidence of further metastatic disease and, therefore, treatment in the form of androgen deprivation therapy. The MDT advocated for no further radiotherapy or novel therapy at this stage.

The classical histological features of prostatic adenocarcinoma typically include cribriform proliferation or micro-acinar structures, characterized by nuclear enlargement and prominent nucleoli [17,18]. In contrast, the histology of testicular malignancies varies significantly depending on the specific type of tumor [19,20]. Seminomas are marked by sheets of uniform cells with clear cytoplasm and a characteristic lymphocytic infiltrate. In contrast, non-seminomatous germ cell tumors and non-germ cell tumors exhibit a range of histological features corresponding to their individual tumor subtypes. Immunohistochemical markers commonly used to identify prostate adenocarcinoma include PSA, prostatic acid phosphatase (PSAP), NKX3.1, P504S, and prostein (P501S). In contrast, the immunohistochemical markers for testicular malignancies vary widely depending on the tumor type, with placental alkaline phosphate (PALP), AFP, and beta-human chorionic gonadotropin (β-HCG) being the most frequently used markers [19,20]. In our case, histological examination revealed extensive infiltration of the testicular tissue by adenocarcinoma, characterized by cribriform glandular structures with round nuclei and prominent nucleoli. Immunohistochemical analysis demonstrated strong positivity for PSA, moderate patchy expression of NKX3.1, and focal strong positivity for chromogranin and synaptophysin. These findings confirmed the diagnosis of metastatic prostate adenocarcinoma, allowing the timely initiation of an appropriate treatment regimen.

The MDT approach was pivotal in diagnosing testicular single-site metastasis. The urologist managed the hydrocele surgery, addressed complications, and performed the inguinal orchidectomy, collaborating with the pathologist, radiologist, and oncologist to confirm the diagnosis. The oncologist monitored the patient’s PSA, revisited imaging after the testicular metastasis diagnosis, and developed the ongoing management plan. The radiologist reviewed prior and new imaging, contributing to MDT discussions. The pathologist identified prostate cancer metastasis on orchidectomy slides, confirmed by additional tests and correlation with the patient’s history.

## Conclusions

This case underscores the complexity and diagnostic challenges associated with rare metastatic presentations of prostatic adenocarcinoma. It emphasizes the importance of thorough follow-up and the need to reassess initial benign diagnoses as the clinical picture evolves. Furthermore, this case serves as a critical reminder of the potential for atypical presentations and metastases in common malignancies such as prostate cancer. Therefore, we highlight the necessity of a multidisciplinary approach in formulating and implementing a comprehensive management plan for such patients.

## References

[1] Smelzo S, Mantica G, Lucianò R (2022). Prostate cancer testicular metastasis: are they underestimated? Case report and analysis of the literature. Urologia.

[2] James ND, Tannock I, N'Dow J (2024). The Lancet Commission on prostate cancer: planning for the surge in cases. Lancet.

[3] Sekhoacha M, Riet K, Motloung P, Gumenku L, Adegoke A, Mashele S (2022). Prostate cancer review: genetics, diagnosis, treatment options, and alternative approaches. Molecules.

[4] Gibas A, Sieczkowski M, Biernat W, Matuszewski M (2015). Isolated testicular metastasis of prostate cancer after radical prostatectomy: case report and literature review. Urol Int.

[5] Bergengren O, Pekala KR, Matsoukas K (2023). 2022 update on prostate cancer epidemiology and risk factors-a systematic review. Eur Urol.

[6] Jain S, Gupta A, Ghosh A (2024). Testicular metastasis in carcinoma prostate: beyond the expected. Cancer Ther Oncol Int J.

[7] Shinn BJ, Greenwald DW, Ahmad N (2015). Unilateral testicular metastasis of low PSA level prostatic adenocarcinoma. BMJ Case Rep.

[8] Maibom SL, Jacobsen A, Hansen RB, Brasso K (2017). Isolated testicular prostate cancer metastasis. Scand J Urol.

[9] Herrera Ortiz AF, Rojas J, Yepes MM, Quiroz Alfaro AJ (2024). Isolated testicular metastasis in a patient with prostate cancer. BMJ Case Rep.

[10] Bonetta A, Generali D, Corona SP, Cancarini G, Brenna SG, Pacifico C, Roviello G (2017). Isolated testicular metastasis from prostate cancer. Am J Case Rep.

[11] Mamaliga T, Obando JA, Liu Y (2023). Testicular metastasis from prostate cancer demonstrated on PSMA PET/CT. Clin Nucl Med.

[12] Gupta N, Dey S, Verma R, Belho ES (2020). Isolated testicular metastasis from prostate cancer detected on Ga-68 PSMA PET/CT scan. Nucl Med Mol Imaging.

[13] Bhasin SD, Shrikhande SS (1990). Secondary carcinoma of testis - a clinicopathologic study of 10 cases. Indian J Cancer.

[14] Waisanen KM, Osumah T, Vaish SS (2017). Testicular metastasis from prostatic adenocarcinoma presenting as recurrent epididymo-orchitis. Urology.

[15] Longoria Porras J, Jiménez Villa A, Maldonado ME (1965). A case of adenocarcinoma of the prostate (encephaloid or medullary type) with intrapelvic metastasis and right hydrocele (Article in Spanish). ME Rev Mex Urol.

[16] Gandaglia G, Abdollah F, Schiffmann J (2014). Distribution of metastatic sites in patients with prostate cancer: a population-based analysis. Prostate.

[17] (2024). Metastatic cancer: when cancer spreads. https://www.cancer.gov/types/metastatic-cancer.

[18] Leslie SW, Soon-Sutton TL, R I A, Sajjad H, Skelton WP (2023). Prostate cancer. StatPearls [Internet].

[19] Leman ES, Gonzalgo ML (2010). Prognostic features and markers for testicular cancer management. Indian J Urol.

[20] Rajpert-De Meyts E, Aksglaede L, Bandak M (2000). Testicular cancer: pathogenesis, diagnosis and management with focus on endocrine aspects. Endotext [Internet].

